# Effects of COVID-19 Illness and Vaccination Infodemic Through Mobile Health, Social Media, and Electronic Media on the Attitudes of Caregivers and Health Care Providers in Pakistan: Qualitative Exploratory Study

**DOI:** 10.2196/49366

**Published:** 2024-09-04

**Authors:** Abdul Momin Kazi, Nazia Ahsan, Rawshan Jabeen, Raheel Allana, Saima Jamal, Muhammad Ayub Khan Mughal, Kathryn L Hopkins, Fauzia Aman Malik

**Affiliations:** 1 Aga Khan University Karachi Pakistan; 2 Sabin Vaccine Institute Karachi Pakistan; 3 University of Texas Southwestern Medical Center Dallas, TX United States

**Keywords:** infodemics, mHealth, social media, electronic media, Pakistan, vaccination, misinformation, infodemiology, mobile phone

## Abstract

**Background:**

The COVID-19 pandemic has had a significant impact on different countries because of which various health and safety measures were implemented, with digital media playing a pivotal role. However, digital media also pose significant concerns such as misinformation and lack of direction.

**Objective:**

We aimed to explore the effects of COVID-19–related infodemics through digital, social, and electronic media on the vaccine-related attitudes of caregivers and health care providers in Pakistan.

**Methods:**

This study employs a qualitative exploratory study design with purposive sampling strategies, and it was conducted at 3 primary health care facilities in the province of Sindh, Pakistan. Seven focus group discussions with health care providers and 60 in-depth interviews with caregivers were conducted using semistructured interviews through virtual platforms (ConnectOnCall and Zoom). Transcripts were analyzed through thematic analysis.

**Results:**

Our study reveals the pivotal role of electronic media, mobile health (mHealth), and social media during the COVID-19 pandemic. Four major themes were identified: (1) sources of information on COVID-19 and its vaccination, (2) electronic media value and misleading communication, (3) mHealth leveraging and limitations during COVID-19, and (4) social media influence and barriers during COVID-19. Health care providers and caregivers reported that the common sources of information were electronic media and mHealth, followed by social media. Some participants also used global media for more reliable information related to COVID-19. mHealth solutions such as public awareness messages, videos, call ringtones, and helplines promoted COVID-19 prevention techniques and vaccine registration. However, the overwhelming influx of news and sociobehavioral narratives, including misinformation/disinformation through social media such as WhatsApp, Facebook, and Twitter, were found to be the primary enablers of vaccine-related infodemics. Electronic media and mHealth were utilized more widely to promote information and communication on the COVID-19 pandemic and vaccination. However, social media and electronic media–driven infodemics were identified as the major factors for misinformation related to COVID-19 and vaccine hesitancy. Further, we found a digital divide between the urban and rural populations, with the use of electronic media in rural settings and social media in urban settings.

**Conclusions:**

In a resource-constrained setting like Pakistan, the usage of mHealth, social media, and electronic media for information spread (both factual and mis/disinformation) related to COVID-19 and its vaccination had a significant impact on attitudes toward COVID-19 vaccination. Based on the qualitative findings, we generated a model of digital communications and information dissemination to increase knowledge about COVID-19 and its prevention measures, including vaccination, which can be replicated in similar settings for other disease burdens and related infodemics. Further, to mitigate the infodemics, both digital and nondigital interventions are needed at a larger scale.

## Introduction

The COVID-19 pandemic has had a deleterious impact on health care systems, economies, and societies globally [1]. This impact has been exacerbated by the resulting COVID-19 infodemic or accompaniment of too much information, including false or misleading information, also occurring in this new digital age of information sharing. With more than 3 billion digital media users globally, digital media has become the key source of information and communication, particularly during a crisis, creating a digital pandemic of information disseminated in multiple forms, regardless of the legitimacy of the sources [2-4]. The downstream effects are confusion, low COVID-19 vaccine confidence, vaccination refusal, and other poorly informed health behavior–related decision-making [5,6]. The most severe consequences are evident in low- and middle-income countries (LMICs) and among marginalized communities [7], where trust in and exposure to official health information sources are comparatively low [8] and there are low health literacy levels, poor health care infrastructure, and less resources [9].

Amid experienced disruptions to health care associated with the COVID-19 pandemic and infodemic, an opportunity also arose. With the advancement of information and technology, digital health played a critical role in COVID-19 response, advocacy, and mobilization [10]. Specifically, digital media assisted in disseminating correct information through mobile health (mHealth)—mobile wireless technologies for public health. These innovations are an integral part of eHealth, which include the cost-effective and secure use of information and communication technologies in support of health and health-related fields, social media–promoted public health initiatives, and electronic media–raised awareness, and these encouraged preventative measures (eg, hand hygiene, vaccine uptake) [11].

During the COVID-19 pandemic, many countries developed mHealth apps to assist with the identification of prevalent symptoms for self-assessment, implementing contact tracing, disseminating information, minimizing exposure, and reducing face-to-face interaction between patients and health workers [12]. A WhatsApp chatbot app in South Africa used machine learning technology to provide free, automated responses to user queries on COVID-19, relating to travel advice, recent statistics, symptoms, and debunking of myths and misinformation [13]. Facebook groups were utilized by most health care professionals during the pandemic to discuss and integrate real-time experiences in COVID-19 treatment [14]. Data visualization dashboards enabled data-driven infographics representing global-to-local pandemic-related statistics, which allowed for the public and researchers to comprehend and track the pandemic in real time [15]. Additionally, social media channels were used to inform citizens about pandemic-related government response efforts and updates such as the national WhatsApp channel established in Singapore during the COVID-19 surge [16].

**Figure 1:**
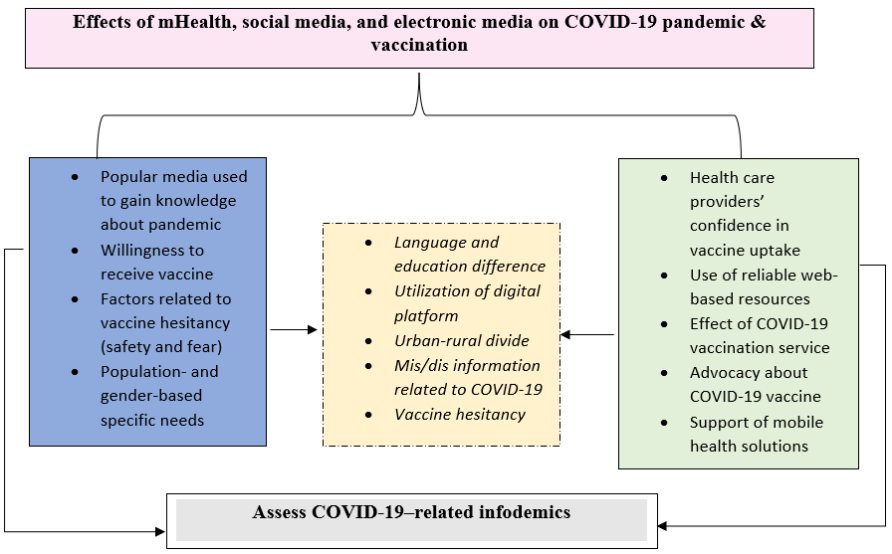
Effects of mobile health, social media, and electronic media during the COVID-19 pandemic. mHealth: mobile health.

Based on the described initiatives above during the COVID-19 pandemic, we have developed a comprehensive framework highlighting the key roles of various digital platforms. This framework highlights how digital tools played diverse and complementary roles in pandemic management—from disseminating information and assessing symptoms to real-time data tracking and communication. These tools were pivotal in proactive intervention, personalized guidance, knowledge exchange among experts, data-driven decision-making, and fostering community resilience through amplified public health messaging and grassroots initiatives (Figure 1).

Pakistan has faced challenges related to vaccine hesitancy and low vaccine acceptance in the past. Pakistan has a history of vaccine-preventable disease outbreaks due to various factors, including misinformation, cultural beliefs, lack of awareness, and mistrust in vaccines. One notable example is the polio eradication efforts in Pakistan. Despite considerable progress made globally, Pakistan has remained one of the few countries where polio cases continue to be reported [17]. Further, Pakistan has diverse cultural and linguistic backgrounds, where different regions may have unique sociodemographic factors that influence vaccine acceptance and hesitancy [18]. Moreover, the COVID-19 pandemic has brought about new challenges and concerns regarding vaccine acceptance globally. It is essential to examine the role of digital media and mHealth interventions in vaccine acceptance, as uptake differs across regions in Pakistan. Factors such as regional beliefs, levels of trust in digital media sources, and access to health care information may vary, leading to different outcomes in vaccine-related decision-making. By exploring these regional differences, we can provide valuable insights into the role of digital health interventions in addressing vaccine hesitancy in diverse settings. It is vital that these digital health interventions continue to be developed to harness social media for the public good and to increase trust in vaccines and vaccination, especially within LMICs like Pakistan. Thus, we explored the effects of mHealth, social media (eg, Facebook, Twitter, Instagram), and electronic media (ie, television and radio) during the COVID-19 pandemic and its association with COVID-19 vaccination and childhood routine immunization acceptance and uptake among parents and child caregivers and health care providers (HCPs) in a resource-constrained setting like Pakistan.

## Methods

### Study Design, Setting, and Population

**Figure 2:**
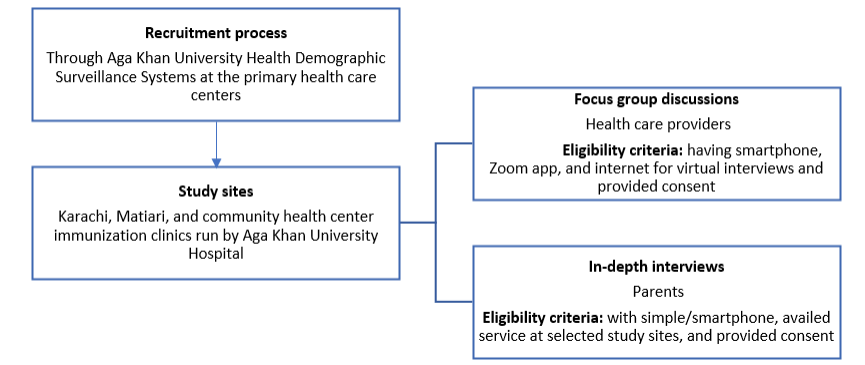
Flowchart depicting participant enrollment.

This exploratory qualitative research was employed to assess the role of infodemics through mHealth, electronic media, and social media in Pakistan to explore the unique experiences and insights of populations, which may be overlooked in quantitative studies [19,20]. This study was implemented between May 2020 and August 2021 at 3 sites in Pakistan: the periurban Aga Khan University (AKU) Health Demographic Surveillance System at primary health care centers of Ali Akbar Shah Goth and Bhains Colony, the rural Sindhi district of Matiari, and the community health center (CHC) vaccination clinic at the AKU hospital in urban Karachi. The 3 selected sites in Pakistan were chosen to represent diverse populations and to capture unique socioeconomic and cultural factors influencing vaccine acceptance and hesitancy. The vaccination center at the periurban sites targeted low-to-middle and low-income populations. The rural area represented a low-income population, and the vaccination center at AKU hospital (urban site) represented both high-income and low-to-middle-income populations. This approach ensured a comprehensive understanding of vaccination behaviors by assessing the role and impact of mHealth, social media, and electronic media on vaccine-related attitudes and behaviors across different socioeconomic contexts. Focus group discussions (FGDs) were implemented among health care workers (doctors, nurses, pharmacists, lady health visitors, vaccinators, lady health workers, and community health workers) in Karachi and Matiari who worked in selected centers, and in-depth interviews (IDIs) were conducted with parents/caregivers of children aged <1 year. Figure 2 shows the flowchart of participant recruitment.

### Ethics Approval

Ethics approval for this study was obtained from the ethics review committee of AKU (2020-5316-14620).

### Inclusion Criteria and Sampling Approach

We used purposive sampling for recruiting HCPs, and convenient sampling was performed for parent selection. Parents or child caregivers were eligible for IDIs if (1) they had at least one child younger than 1 year and (2) their telephonic contact number was listed in either the registry maintained by the AKU hospital and its associated centers at the study sites or provided by community health workers affiliated with the study sites. The IDI was conducted with 1 caregiver at a time. HCPs such as doctors, nurses, pharmacists, lady health visitors, vaccinators, and community health workers at each of the 3 study sites were eligible as FGD participants; 6-8 participants were included in the FGDs. The parents/caregivers and HCPs who did not provide consent were excluded from this study.

### Study Framework and Tool

Semistructured qualitative interview guides were developed for both IDI and FGD data collection in English and local Urdu language with the help of a literature review [21-23]. The main topics discussed were access to electronic and social media among diverse populations, barriers and perceived challenges, caregivers/parental concerns about vaccine safety, understanding of content available on digital media, and word-of-mouth communication within the community. Moreover, HCPs were more focused on factors associated with COVID-19 fear and dis/misinformation in the data, which may lead to vaccine hesitancy. We used the process defined in Figure 2 to gather data.

### Data Collection and Management

A team of researchers from AKU designed and piloted the research tools and trained qualitative research staff for conducting FGDs and IDIs. Due to the ongoing pandemic, all data collection was conducted remotely as per COVID-19 standard operating procedures (SOPs). Seven FGDs with HCPs and 60 IDIs with caregivers were conducted using semistructured interviews through virtual platforms (ConnectOnCall and Zoom) till the point of saturation. Two alternative methods were employed to acquire data: IDIs of 40-60 minutes were conducted telephonically, while FGDs of 60-90 minutes with HCPs were conducted using the Zoom videoconferencing platform. After obtaining verbal informed consent for participation, the respondents affiliated with all 3 study sites were given an overview of the study objectives, and parents/caregivers were provided training or guidance on how to attend virtual interviews on phone, which was helpful for individuals who were not tech-savvy or had limited experience with virtual communication. HCPs were debriefed about the Zoom platform and its related features to avoid any hindrance while interviews were being conducted. Data were recorded on the phone and a Zoom password-protected device.

### Data Analysis

Thematic analyses were adopted as part of the qualitative study. Each audio recorded Sindhi and Urdu-spoken IDIs, and FGDs were transcribed and translated to English. The English transcripts were then assessed and coded separately and connected to text fragments that reflected crucial user perspectives. The data were subsequently organized into thematic categories by looking for topics and then reviewed, defined, and named. We ensured trustworthiness of the data analysis by using Lincoln and Guba guidelines [24] to reduce researcher bias. Further, all discrepancies were resolved after team discussion; codes were finalized to generate the major themes emerging through FGDs and IDIs. The team then contrasted the themes before more targeted coding, focusing on ideas linked to mHealth, social media, and electronic media.

## Results

### COVID-19–Related Infodemics

The key findings with regard to the COVID-19–related infodemic are listed in Table 1.

**Table 1:** COVID-19–related infodemic.

Subtheme	Key findings
Electronic media infodemic	Overselling of news Both factual and false information Fabricated news of COVID-19 and vaccination Influence of negative political narratives Anxiety due to exaggerated breaking news
Social media infodemic	Negative impact of videos (burial of dead due to COVID-19) Overhype of content, creating fear of contracting infection Sharing of conflicting posts (audios and videos) False information through nonmedical professionals Myths about adverse effects of COVID-19 vaccine: brain damage, infertility, and death
Mobile health infodemic	Lack of pandemic-related information in rural populations Limited access to mobile phone Parents were unable to understand information about COVID-19 information through ringtones Reluctant about sharing personal identification number required for vaccination registration

### Access to Information Through Print, Electronic, and Digital Media

HCPs and caregivers shared that COVID-19–related information was commonly disseminated through print media, mHealth, and social and electronic media. The HCPs of the urban site reflected their beliefs about the use of the medium as authentic, as mobile phones (WhatsApp) were easily accessible and conveniently used. Moreover, HCPs also used Facebook to access information from the official pages of World Health Organization, Centers for Disease Control, and other international websites. Although a few caregivers also used international media for more reliable information related to COVID-19, caregivers in rural areas showed concern about the lack of media access and limited services on mobile networks.

…I get credible information from Google. We also access COVID-related information from Facebook pages of WHO, CDC and other international health organizations and they (HCPs) received official updated information through WhatsApp by senior management.

FGD, HCP from Karachi

…Women do not own mobile phones. Usually, they are used by males in our community.

FGD, HCP from Matiari

…COVID-19–related SMS is important in those areas where there are not many ways of information like small cities and villages, where there is no internet available.

IDI, parent/caregiver from CHC

### mHealth: Perceived mHealth Solution Usefulness and Challenges During COVID-19

During the pandemic, SMS text message–based mobile interventions were used to promote COVID-19 prevention techniques and register the general population for COVID-19 vaccination at both urban and rural sites. These messages tried to influence public behaviors such as SOPs related to COVID-19 prevention, follow-up appointments, and immunization advantages. Caregivers endorsed these government services. Caregivers shared that they received messages for the registration for COVID-19 vaccination of people older than 60 years. HCPs stated that conducting vaccination registration with national identity card numbers was a good initiative and felt an automated system that shared reminder messages would help reduce the defaulters.

…Public service-SMS are beneficial in spreading awareness about COVID-19 pandemic, SOPs, and getting vaccinated against COVID-19.

FGD, HCP from Matiari

…The public service-SMS is about COVID-19 vaccine registration, if your age is above 60 then you can get your vaccines, for registration send your CNIC number to 1166.

IDI, parent/caregiver from CHC

Urban caregivers were suspicious whenever the national identity card number was asked for vaccination or other treatments, as a circulating rumor via Twitter stated that these local data were being gathered for use by foreign agencies such as the United States or China.

…They ask for your NIC number when you go to get your COVID-19 test done. There were such gossips making rounds that your data will be sold to China or USA or on Twitter, like China and USA will control the world.

IDI, parent/caregiver from CHC

### Social Media

#### Community Access to Social Media and Information

HCPs perceived that social media, including WhatsApp, YouTube, and Facebook, were used as a positive channel for circulating updated information during the COVID-19 pandemic. However, they also shared that vaccine hesitancy increased among the general population because of the social media infodemic. Caregivers in urban areas reported using social media such as Facebook and the internet to learn about COVID-19 prevalence and news updates, but those in rural areas reported that they never used social media.

…Social media (Facebook, WhatsApp, Instagram, YouTube) is the first source of information about COVID.

FGD, HCP from CHC Karachi

#### Conflicting News Related to COVID-19 and Its Spread Through Social Media

Caregivers shared that the news circulating via social media were largely negative and fabricated, creating fear among the general population in both settings. Participants felt social media was full of opinionated people who deliberately post to impose their personal beliefs on the public.

…Things shared on social media are completely meaningless. Everyone became a doctor and began to demonstrate their competence. Social media is rife with stories about people becoming unwell after receiving their first dose of vaccination. People are skeptical of the COVID-19 vaccine.

IDI, parent/caregiver from Matiari

…We get awareness through these messages, but sometimes rumors are being spread through social media posts, so we need to check the information being shared and look at its sources and determine if the messages are credible and true or false

FGD, HCP from Karachi

#### COVID-19 Pandemic and Vaccine Rumors Spread Through Social Media

According to these caregivers, the news about vaccine-related adverse events were shared by trusted community members such as friends and relatives on platforms such as WhatsApp and Facebook. This information spread quickly, leading to skepticism about the safety and efficacy of COVID-19 vaccines. A few caregivers reported that the news on social media shared that COVID-19 immunizations caused brain stroke/clotting and that this news led to vaccine hesitancy.

…People got hesitant to receive the COVID-19 vaccination after reading on social media that it causes brain coagulation.

IDI, parent/caregiver from CHC

…Then there were rumors that so many people died or got infected after getting the vaccine.

FGD, HCP from Matiari

One of the significant challenges regarding news was the uncertainty of the delivery of accurate information and its understanding among the population. Caregivers shared multiple rumors regarding COVID-19 immunization such as it caused infertility in unmarried people and congenital malformations in children of vaccinated pregnant women and increased mortality rate among vaccinated people.

…After getting COVID-19 vaccine, you might become infertile and unable to conceive. There were rumors that many people died or got infected after COVID-19 vaccine.

FGD, HCP from Matiari

### Electronic Media

#### Role of Electronic Media Platforms During the COVID-19 Pandemic

Electronic media was the main source of information for the rural population. The caregivers reported that electronic media had a significant impact on communities and that international media such as the British Broadcasting Corporation (BBC) never fabricated information as compared to the other national news channels. Further, COVID-19 news about morbidity and mortality was telecast as a sensational issue. According to HCPs, there were many erroneous calls and SMS text messages with incorrect information circulating among the caregivers. Nonetheless, the information was validated by HCPs before passing it to others. Further, they double-checked every news item from official sources before sending it to others.

…In my opinion, news that is broadcasted on BBC is not fabricated. So, media should encourage more programs and talk shows on COVID-19 prevention and its vaccination rather than sensational news on morbidities and mortalities.

IDI, parent/caregiver from CHC

…Public service messages are important in creating awareness about COVID-19 pandemic only if they are sent from a relevant source like official government websites or numbers, then you know that this is a credible bit of information.

FGD, HCPs from CHC Karachi

#### COVID-19 Vaccine Hesitancy and Misleading Information Spread Through Electronic Media

HCPs expressed that sometimes nonmedical personalities shared their opinions on the news, which confused the population and negatively influenced other individuals’ vaccination behavior. One of the caregivers in the urban site expressed that he felt worried and stressed after receiving COVID-19–related news. The rural population expressed comparable concerns about news-related anxiety. Caregivers believed that both positive and negative news broadcasted on television and other media influenced the population’s mental health.

…I heard Provincial Government’s statement on television that if you want to get this vaccine then get it at your own risk. If government officials continue to talk like this, it will create a negative impact regarding vaccines in the minds of the people. Educational people will also have uncertainties and concerns because of it.

FGD, HCP from Matiari

## Discussion

### Principal Findings

The COVID-19 pandemic has had both health and societal implications, which were not comparable to any recent event in global history. Social distancing during COVID-19 had a great impact, which led to an increase in the widespread use of digital platforms for acquiring the latest updates and COVID-19–related communication. Our qualitative study highlights many critical issues based on the usage of mHealth and social and electronic media related to the COVID-19 pandemic and vaccine coverage. The primary findings of our study indicate that according to HCPs and caregivers, electronic media and mHealth were used more broadly to promote COVID-19 pandemic-related information and communication. However, the social and electronic media–driven infodemics and urban-rural divide posed major obstacles. Further, digital media not only enabled individuals to quickly access and share information on disease spread and emerging health policy changes but also provided access to resources, community engagement, and misinformation.

Our study shows electronic media as the most effective tool for communicating health-related messages such as awareness of COVID-19 SOPs and vaccination to reduce the general population’s fear of acquiring infection. Electronic media provided the most reliable information related to the pandemic and hence became the most dependable medium to receive information, especially for the rural population. Electronic media was the primary source for filling the information gap among the general populations in Pakistan. However, there was an element of misinformation and disinformation in the news broadcasts related to the COVID-19 pandemic, which created confusion and misled the general population. This finding was similar to findings in Korea [25], wherein using electronic media to access vaccine-related information had a positive impact on the population’s vaccination-related decision-making as well as higher perceived benefits of the COVID-19 vaccine and greater trust in the government to address vaccine hesitancy. Further, an Italian study highlighted the importance of health information transmitted to raise awareness among individuals through radio, television, and journalistic communications [26]. However, despite motivating the population to reduce their fear and stress related to the COVID-19 pandemic, television broadcasts commonly used complicated language, technical jargon, and disseminated debates, thereby creating more confusion about the situation [27].

Our study explores the usefulness of mHealth as a communication media to disseminate information and support the population and HCPs during the pandemic. Participants in our study reported that mHealth strategies such as ringtone messages disseminated COVID-related awareness and preventive strategies and were actively used as a communication medium about public health awareness messages during different phases of the pandemic in the overall population. Most people could understand the ringtone because it was played in local languages. A similar strategy has been recommended to replace entertainment and religious ringtones with health promotion, particularly for LMICs [28]. Our findings also revealed that HCPs approached the COVID-19 dashboard and national and international resources for credible sources of information, which motivated them to deliver services and enabled vaccination uptake. SMS text messaging also played a critical role in the web-based registration for the COVID-19 vaccine. Moreover, reminders were automatically forwarded to the general population for missing doses of COVID-19 vaccine. Another study revealed that sending SMS text messages, emails, and postal messages enhanced influenza vaccination appointments [29]. According to our study participants, SMS text message–based vaccine registration was significantly accepted by the population and could potentially be further expanded to other adult and child vaccinations in Pakistan and other LMICs.

During the pandemic, the role of social media was widespread in providing information about COVID-19 and its vaccines through Facebook, Twitter, and WhatsApp. However, social media also propagated misinformation and disinformation related to COVID-19, hence introducing the phenomena of infodemics. In Pakistan, WhatsApp was widely used for disseminating pandemic-related information. Similarly, a study conducted in the United Arab Emirates reported WhatsApp as being mostly used to acquire COVID-19–related information [30].

We found that social media news and its content contributed to increasing COVID-19–related anxiety and stress among the population, as stated by HCPs. In our study, political tweets received more engagement, as leaders’ tweets had an enormous influence on the public, creating confusion and misconception. Another study showed that leaders’ tweets had higher interactions in high-income nations like the United Kingdom as well, and the leader’s tweets had a stronger impact on the general population, creating misinterpretation, which ultimately impacted vaccine uptake [31].

During the pandemic, information regarding vaccine adverse effects and misinformation was circulating on television and social media, adding fear and uncertainty among the population, and leading to a decline in vaccine acceptance. Our study participants also reported infertility, brain stroke, and death-related misinformation related to COVID-19 vaccines. Our findings are consistent with those of a US-based study, which reported similar misinformation falsely correlating COVID-19 vaccination with infertility and population growth control, electronic tattooing or microchipping individuals for global surveillance, and autism, which resulted in low vaccine uptake by the general population overall [32]. Thus, our HCP participants emphasized that lack of news verification and monitoring and incorrect information dissemination resulted in confusion and vaccine hesitancy among caregivers. This qualitative study provides insights that lack of technological literacy in the rural population was a hindrance in adopting mHealth interventions in the rural setting. Our study participants were not aware of or did not report about the official COVID-19 Government of Pakistan website. Besides that, the rural population, especially women, did not own personal mobile phones and were unfamiliar with its usage due to lack of technological literacy. However, in a rural setting in India, where the literacy rate of women was 40.35%, nearly 85% of the rural illiterate women were found to be using a mobile phone without necessarily owning it. It was their quickest means of communication and receiving information [33].

**Figure 3:**
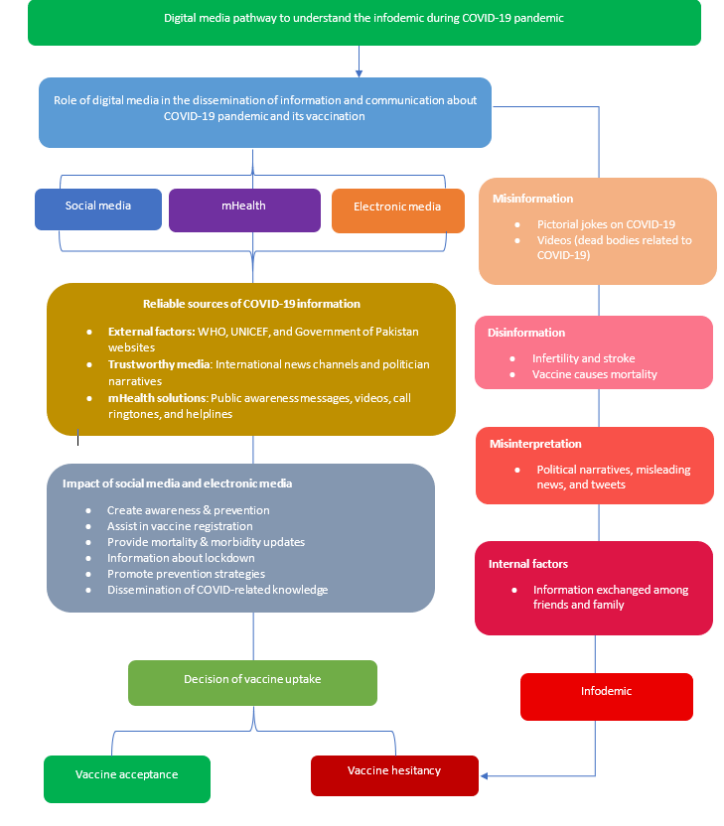
Infodemic pathway of digital media. mHealth: mobile health; UNICEF: United Nations International Children's Emergency Fund; WHO: World Health Organization.

The digital pathway model that we conceptualized is based on national policy interventions (SMS text message–based interventions, caller tunes, vaccine registration through SMS text messages) and the responses of our research participants, which enabled them to communicate and disseminate information during the COVID-19 pandemic and assisted them in the fight against pandemic-related infodemics (Figure 3). We have discussed the diversity of mHealth interventions and their usage along this pathway. This framework is required to understand how digital methods might be integrated into COVID-19 control efforts and to aid in future pandemic preparedness (Textbox 1).

We examined platforms commonly used in misinformation campaigns in our settings, such as mHealth and social and electronic media. The flowchart’s development revealed a critical insight into how different media play distinct roles in supporting the spread of misinformation via different paths. Our model has crucial information for policy makers who want to combat the phenomena of infodemics and the digital divide. Policy makers at the government level across the world need to strategize social and legal regulatory frameworks to curb the spread of misinformation and disinformation in digital media.

**Digital methods that were integrated into COVID-19 control efforts and that can aid in future pandemic preparedness.**

- Electronic media provided coverage to the overall population through advertisements, news, and broadcasts, minimizing the digital communication gap between the urban and rural populations.
- Mobile health–based initiatives such as SMS text messages, calls, helplines, and caller ringtones were the sources of public awareness and information, which were enhanced using local languages. Other external media such as World Health Organization websites and news channels were used as reliable resources.
- Social media enabled wide access to information, particularly in the urban population, and dissemination of misinformation and disinformation about vaccines and pandemics.
- Electronic media, mobile health, and social media were the major modes of communication and dissemination during the pandemic, which promoted COVID-related standard operating procedures and vaccine acceptance. However, these same media also became the source of infodemics, leading to misguided information and vaccine hesitancy.

### Study Limitations

Our results were based on participants visiting primary health care centers operated by private health entities with limited opening hours, staffing, and financial resources, constituting a significant study limitation. Further, the findings related to COVID-19 vaccine hesitancy cannot be generalized to other groups with different social and cultural backgrounds of other provinces. Thus, quantitative research is required to mitigate infodemics in larger populations.

### Implications and Recommendations

The COVID-19 pandemic has necessitated the use of digital platforms for communication and information dissemination, with electronic media, mHealth, and social media playing crucial roles. Electronic media emerged as an effective tool for promoting COVID-19–related information and reducing fear among the general population. However, it also became a source of confusion causing mis/disinformation. Further, to improve public health interventions, precise and accurate information that pertains to World Health Organization or Centers for Disease Control guidelines should be allowed to be aired on televisions and media so that they can function as a bridge for people to connect with health officials and local governments for assistance, and collaborations between government and media outlets can establish guidelines for responsible reporting [34]. mHealth strategies such as SMS text message notifications, ringtone messages, and mobile apps proved useful in raising public awareness, especially in rural areas. Policy makers should invest in user-friendly mHealth platforms to ensure that timely and accurate information reaches individuals, particularly in low-income settings. Lastly, social media platforms also played a pivotal role in providing pandemic-related information, which was not previously seen on that scale, but they unfortunately also contributed toward infodemics. Regulatory bodies should develop frameworks to mitigate misinformation on social media, including collaboration with social media companies, monitoring mechanisms, and awareness campaigns. These frameworks should include collaboration with platforms for fact-checking and prioritizing authoritative sources, real-time monitoring using artificial intelligence, public awareness campaigns promoting critical thinking, and regulatory oversight ensuring transparency and accountability [35]. Addressing the challenges posed by infodemics and the digital divide requires enhancing information verification, promoting digital media literacy in rural areas, and strengthening public health communication through partnerships with influencers and HCPs. By implementing these strategies, policy makers can improve information dissemination, mitigate misinformation, and enhance future pandemic preparedness efforts.

### Conclusion

This study proposes the implementation of a communication pathway focused on diseases and pandemics to be integrated into a national digital policy in resource-constraint setups, including Pakistan. This would enhance the country’s readiness to respond to health crises and boost public awareness and comprehension of these matters. Although digital media emerged as the primary source of information during the pandemic, it also contributed to misinformation and disinformation, causing infodemics. It is therefore essential to comprehend the sources and content of information within each digital medium element that triggers infodemics. LMICs are more vulnerable to infodemics because they have limited access, awareness, and lower literacy levels to comprehend and evaluate health-related information. Our research findings further revealed a digital divide between urban and rural populations, resulting in digital inequalities. To address these challenges, both digital and nondigital solutions must play a vital role. Moreover, credible information must be widely disseminated from trustworthy sources, verified by subject matter experts, and tailored to fit the local context. Lastly, training programs on the usage of digital media, dissemination strategies, and information reliability need to be conducted, particularly for HCPs and different settings, especially to reach rural communities.

## Abbreviations

AKU: Aga Khan University
BBC: British Broadcasting Corporation
CHC: community health center
FGD: focus group discussion
HCP: health care provider
IDI: in-depth interview
LMIC: low- and middle-income country
mHealth: mobile health
SOP: standard operating procedure
